# Fusing gene expressions and transitive protein-protein interactions for inference of gene regulatory networks

**DOI:** 10.1186/s12918-019-0695-x

**Published:** 2019-04-05

**Authors:** Wenting Liu, Jagath C. Rajapakse

**Affiliations:** 1 School of Public Health and Management, Hubei University of Medicine, Shiyan, Hubei China; 2 Integrative Biology and Physiology, University of California, Los Angeles, Los Angeles, CA USA; 3 School of Computer Engineering, Nanyang Technological University, Singapore, Singapore

**Keywords:** Gene regulatory network (GRN), Gene expressions, Gaussian mixture model (GMM), Protein-protein interaction networks, Transitive protein-protein interactions

## Abstract

**Background:**

Systematic fusion of multiple data sources for Gene Regulatory Networks (GRN) inference remains a key challenge in systems biology. We incorporate information from protein-protein interaction networks (PPIN) into the process of GRN inference from gene expression (GE) data. However, existing PPIN remain sparse and transitive protein interactions can help predict missing protein interactions. We therefore propose a systematic probabilistic framework on fusing GE data and transitive protein interaction data to coherently build GRN.

**Results:**

We use a Gaussian Mixture Model (GMM) to soft-cluster GE data, allowing overlapping cluster memberships. Next, a heuristic method is proposed to extend sparse PPIN by incorporating transitive linkages. We then propose a novel way to score extended protein interactions by combining topological properties of PPIN and correlations of GE. Following this, GE data and extended PPIN are fused using a Gaussian Hidden Markov Model (GHMM) in order to identify gene regulatory pathways and refine interaction scores that are then used to constrain the GRN structure. We employ a Bayesian Gaussian Mixture (BGM) model to refine the GRN derived from GE data by using the structural priors derived from GHMM. Experiments on real yeast regulatory networks demonstrate both the feasibility of the extended PPIN in predicting transitive protein interactions and its effectiveness on improving the coverage and accuracy the proposed method of fusing PPIN and GE to build GRN.

**Conclusion:**

The GE and PPIN fusion model outperforms both the state-of-the-art single data source models (CLR, GENIE3, TIGRESS) as well as existing fusion models under various constraints.

## Background

Gene regulations describe the interactions among genes during cellular activity. Through regulation, genes orchestrate the level of synthesized mRNA and thereby control the expression of other genes and the rates at which proteins are produced, eventually deciding the state of the cell. Gene expression (GE) microarrays provide quantitative or semi-quantitative data on the cell state at a specific time and condition. By “reverse-engineering” GE data, regulatory interactions among genes can be identified and gene regulatory network can be mapped using computational methods [1, 2].

Vast majority of functional analysis approaches to modelling microarray GE data assume that genes with similar expression profiles have similar cellular functions [3–5]. A molecular pathway is a set of genes that activate together to achieve a specific task and thus share similar expression profiles. In this paper, we use a data-driven method - the model-based clustering - to model genes in distinct pathways. Specifically, we model each pathway as a Gaussian model as it allows modelling correlations among gene expressions in a data-driven way. It is better suited for situations where the prior knowledge of the regulatory pathways is unknown. In addition, because genes naturally participate in more than one regulatory pathway, soft-clustering is used to allowed so that genes can have memberships in multiple pathways. Hence, we adopt the Gaussian mixture model (GMM) on GE data so that different regulatory pathways can be identified.

The rationale behind clustering is that co-expressed genes, i.e., genes in the same cluster are more likely to be functionally related and belong to the same cluster. However, regulatory processes of the genes in a cluster could not necessarily be direct as it could refer to an indirect regulation via proteins, metabolites, or ncRNAs. In cases where two interaction partners are transcription factors or where two proteins are in the same complex, the interactions are direct. In order to identify indirect regulations in GRN, evidences from multiple data sources should be used. For example, medical literature, protein-protein interaction (PPI) data, gene ontology, etc., have all been used to supplement wet lab data in the inference of GRN [6–9]. When more than one source are available, an essential step is to optimally combine evidences from multiple sources to derive a coherent GRN [10–12].

Since proteins are products of genes, protein interactions provide useful evidence for gene regulation. PPIN data have been fused with GE data for GRN inference in previous studies [13–17]. Most of these works considered only binary links of PPIN: if the link is consistent with the predicted edge from GE, the link is accepted as a true regulation. This approach throws away valuable information, so an accurate quantitative scoring scheme is needed to evaluate consistency between PPIN and gene regulation. On the other hand, existing PPINs are sparse and many real protein interactions are missing in current PPIN databases. Suggested by previous PPI prediction works [18, 19], there exist a large number of interactions between proteins in complexes, which have not yet been observed or recorded in current PPIN. We therefore propose a heuristic to quantitatively extend sparse PPIN by using transitive linkages. We then propose a novel way to score protein interactions by combining topological properties of extended PPIN and correlations of GE. Our experiments demonstrate that transitive protein interactions indeed play an important role in predicting protein interactions. We fuse the extended PPIN scores with GE data, using a Gaussian hidden Markov model (GHMM) to identify gene regulatory pathways, which are found to more consistent with PPIN than those produced by GMM. We further refine PPIN confidence scores by including gene interaction scores from GHMM, which makes the PPIN score more consistent with the existing GRN.

**Fig. 1:**
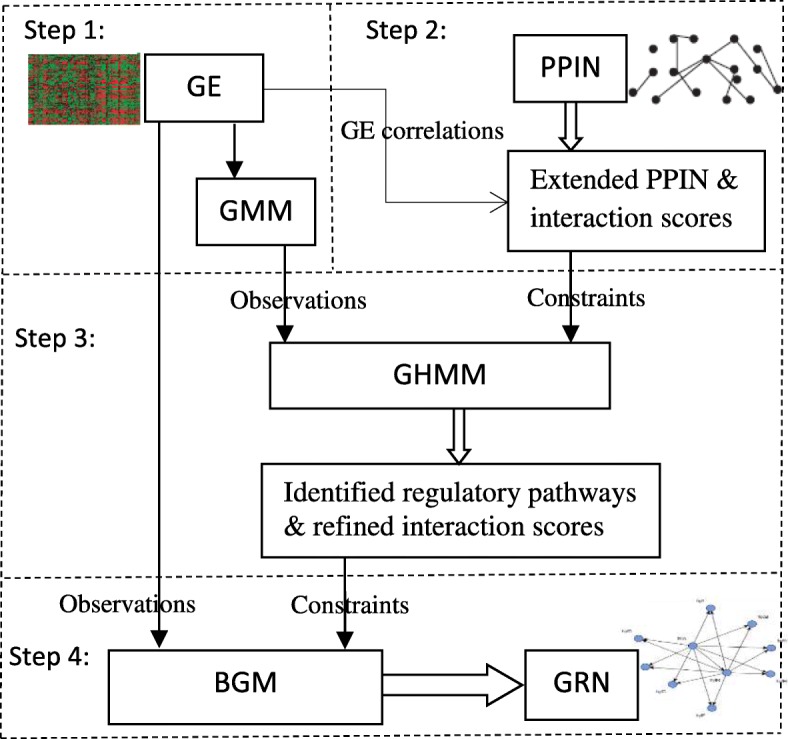
Overall Model. Step 1: A Gaussian mixture model (GMM) is used to soft-cluster gene expression (GE) data. Step 2: A heuristic is proposed to quantitatively extend the sparse protein-protein interactions by using transitive linkages. A novel way is then proposed to score protein interactions by combining topological properties of extended protein-protein interaction network (PPIN) and GE correlations. Step 3: A Gaussian Hidden Markov Model (GHMM) is used to identify gene regulatory pathways and refine interaction scores, both of which are then used as structural priors to constrain the model of GRN. Step 4: Lastly, the GRN from GE is refined using a Bayesian Gaussian Mixture (BGM) model by including the structural priors derived from Step 3

Since there exists no widely accepted model that universally fits GE data well [20–23], and different models capture different GE properties leading to different or complementary GRN structures [21], fusion of different models should lead to better GRN. The GHMM identifies regulatory pathways and obtain possible interacting genes by considering linear correlations between genes but misses conditional dependencies, i.e., non-linear relations, among genes in the same regulatory pathway. The Bayesian network (BN) model is good at capturing these conditional dependences but suffers from poor computational efficiency. We thus propose a systematic probabilistic framework that fuse these two models and derive coherent GRN closer to biological reality. Specifically, our framework takes a coarse-to-fine approach: GHMM generates regulatory pathways (i.e., a coarse GRN having high coverage) and obtains refined interaction scores, both of which are then used to constrain the GRN structure of a BN model (i.e., the Bayesian Gaussian mixture model). This generates GRN that are of good coverage and high precision. Furthermore, GRN structural constrains help greatly reduce the search space for BGM model, thereby reducing the overall computational complexity. Figure 1 shows the flow chart of our GRN inference process.

## Methods

### Soft-Clustering of GE by Gaussian mixture model

#### Gaussian mixture model

Since gene expression measurement can be viewed as an expression of every gene over all the possible pathways, we employ a Gaussian mixture model (GMM) [24] to describe gene expression (GE) data. For simplicity, we assume that the expression of genes in a pathway follows a Gaussian model, and gene expression data are generated by a finite mixture of underlying probability distribution, that is, by multivariate normal distributions. The key difference between our work and existing GMM hard clustering [13, 24] of GE data lies in our assumption that each gene can participate in multiple pathways. That is, we allow soft-clustering membership for genes so that they can participate in multiple pathways. When one gene participates multiple pathways, it can have soft-clustering membership values for different pathways, representing its contributions of gene expressions in the Gaussian model in different pathways.

Given a gene expression dataset, let $\mathcal {I}=\{i\}_{i=1}^{I}$ denote the set of genes, $\mathcal {L}=\{l\}_{l=1}^{L}$ be the set of pathways, and $\gamma _{i}\in \mathcal {L}$ denote the regulatory pathways in which gene *i* participates, i.e., the pathway assignment for gene *i*. The regulatory pathway assignments of gene set $\mathcal {I}$ is denoted by the set $\Gamma =\{\gamma _{i}\}_{i=1}^{I}$. The pathway assignment variable *γ*_*i*_ follows a multinomial distribution parameterized by vector $\Theta =\{\theta _{l}\}_{l=1}^{L}$, with assignment probabilities *p*(*γ*_*i*_=*l*)=*θ*_*l*_, *θ*_*l*_∈[0,1] where $\sum _{l=1}^{L} \theta _{l}=1$. We assume that the set of weights $\Theta =\{\theta _{l}\}_{l=1}^{L}$ follow a Dirichlet distribution *p*(*Θ*)=*Dir*(*α*_1_,…,*α*_*L*_) with Dirichlet weights having uniform priors.

Given gene expression data $\mathcal {X}=\{x_{i}\}_{i\in \mathcal {I}}$, each instance *i* has *J* continuous-valued attributes $x_{i}=\{x_{ij}\}_{j=1}^{J}$, where *x*_*ij*_ represents the gene expression value measured for gene *i* in experiment *j* out of a total of *J* experiments. We represent each pathway with a Gaussian model wherein the tuple representing the gene expression levels of each experiment (conditioned on a pathway) is a multivariate sample. The probability of observing a given tuple of gene expression levels conditioned on a pathway is *p*(*x*_*i*_|*γ*_*i*_=*l*)∼*N*(*μ*_*l*_,*Σ*_*l*_), where *μ*_*l*_ is the mean vector and *Σ*_*l*_ is the *J*×*J* covariance matrix.

The pathway assignments are determined by $p(\Gamma |\mathcal {X})\propto p(\mathcal {X},\Gamma)$. Assuming independence between genes, the joint distribution over $\mathcal {X}$ and pathway assignments *Γ* is

1 $$ p(\mathcal{X},\Gamma) = \prod\limits_{i=1}^{I} p(x_{i},\gamma_{i}) \\ = \prod\limits_{i=1}^{I} \sum\limits_{l=1}^{L} p(x_{i},\gamma_{i}=l)  $$

#### Learning parameters of the Gaussian mixture model

Expectation Maximization (EM) algorithm [25] is used to estimate the parameters of the joint likelihood in (1). The EM algorithm generates a sequence of parameter approximations that eventually maximizes the observed likelihood. The parameters are initialized to random values. The *t*-th iteration comprises the following two steps.

E-step: Compute the probability of gene {*i*:*i*=1,…,*I*} conditioned on regulatory pathway {*l*:*l*=1,…,*L*} by using current parameter estimates as:

2 $$\begin{array}{@{}rcl@{}} p\left(x_{i}|\mu_{l}^{t},\Sigma_{l}^{t}\right) & \sim & N\left(\mu_{l}^{t},\Sigma_{l}^{t}\right) ;  \\ \tau_{il}^{t}=p(x_{i},\gamma_{i}=l) & = &\frac{\theta_{l}^{t} p\left(x_{i}|\mu_{l}^{t},\Sigma_{l}^{t}\right)}{\sum_{l^{\prime}=1}^{L} \theta_{l^{\prime}}^{t} p\left(x_{i}|\mu_{l^{\prime}}^{t},\Sigma_{l^{\prime}}^{t}\right)}. \end{array} $$

This requires *I*×*L* computations.

M-step: Re-estimate the GMM parameters $\left \{\theta _{l}, \mu _{l}, \Sigma _{l}\right \}_{l=1}^{L}$ for all *L* pathways as:

3 $$ \begin{aligned} \theta_{l}^{t+1} & = \frac{1}{I}\sum_{i=1}^{I} \tau_{il}^{t}; \\ \mu_{l}^{t+1} & = \frac{\sum_{i=1}^{I} \tau_{il}^{t} x_{i}}{\sum_{i=1}^{I} \tau_{il}^{t}}; \\ \Sigma_{l}^{t+1} & = \frac{\sum_{i=1}^{I} \tau_{il}^{t} \left(x_{i}-\mu_{l}^{t+1}\right)\left(x_{i}-\mu_{l}^{t+1}\right)^{\prime}}{\sum_{i=1}^{I} \tau_{il}^{t}}. \end{aligned}  $$

Once EM procedure converges, we accumulate the joint probability of each pair of genes (*i*,*i*^′^) over all the pathways. This gives the pairwise interaction probability of two genes $G_{i,i^{\prime }}$, which measures how likely both genes belong to the same pathway (assuming that genes are mutually independent):

4 $$ G_{i,i^{\prime}} = p\left(x_{i}, x_{i^{\prime}}, \gamma_{i}=\gamma_{i^{\prime}}\right) = \sum\limits_{l=1}^{L} \tau_{il}\tau_{i^{\prime} l}.  $$

where *τ*_*il*_=*p*(*x*_*i*_,*γ*_*i*_=*l*) be the joint probability of gene *i* belonging to the *l*-th regulatory pathway. That is, from genes’ pathway assignments matrix *τ*={*τ*_*il*_}_*I*×*L*_, we obtain the first version of GRN as a multiplication of *τ* and its transpose *τ*^*T*^:

5 $$ G = \tau\times\tau^{T}.  $$

Note that $G = \left \{G_{i,i^{\prime }}\right \}$ consists of the probabilities of gene pairs belonging to the same regulatory pathways. Each entry $G_{i,i^{\prime }}$ denotes the interaction probability of gene *i* and *i*^′^, estimated from GE data.

The number of mixture components *L* can be determined by the component-wise EM algorithm [25] automatically, instead of trying all possible *L*∈[*L*_*min*_,*L*_*max*_] via the time-consuming EM algorithm. The idea is to make use of the non-increasing property of $p(\mathcal {X},\Gamma |\mathcal {M}_{L})$ with respect to *L*, implying that the minimum message length (MML) criterion $\text {MML}(\mathcal {X},\Gamma,\mathcal {M}_{L})$ also decreases with *L*. Starting from *L*=*L*_*max*_, we run the EM algorithm and compute the MML. At the next iteration, *L* is decremented by eliminating the smallest or empty components (setting the smallest *θ*_*l*_ as 0). The E and M steps are repeated to compute an updated MML value at each iteration until $\text {MML}(\mathcal {X},\Gamma,\mathcal {M}_{L})$ converges. In this way, not all values of *L* need to be evaluated, saving unnecessary computations.

### Structural inferences from PPIN

#### Implicit PPI derived from transitivity

Suggested by [18], there exist a large number of interactions between protein complex components that are not yet observed or recorded. Yamada et al. [19] showed that PPIN evolutionary properties, e.g., shortest path, clustering coefficient, give some clues for potential protein interactions. We therefore propose a heuristic to augment protein-interaction networks (PPIN) by assuming transitivity among known protein interactions. The rationale behind this heuristic is also the base for a lot of similarity-based approaches of predicting GRNs, e.g., the relevance networks based algorithms [26] assumed that “if A is similar to B, then A interacts with B”. Hence, “if A is similar to B, and B is similar to C, then A is similar to C, implying that A interacts with C.” As such, we infer implicit interactions based on the first-order transitivity assumptions of protein interactions, i.e., if A interacts with B and B interacts with C, we infer that A interacts with C. Suthram et al. [27] designed methods to assign confidence scores of predicted potential protein interactions from multiple data sources, i.e., by combining GE data, literature, and PPIN data. Inspired by their work, we assign confidence scores of implicit protein-protein interactions (PPI) based only on GE and PPIN data. We use a confidence scoring scheme similar to that of [27]. The novelty of our score lies in its combination of the shortest path score and the Markov clustering score (a graph property) instead of the clustering coefficient.

We extract the shortest path of length $d_{ii^{\prime }}$ from protein/gene *i* to protein/gene *i*^′^ in protein interaction network *P* whose gene set is $\mathcal {I}$ by using Dijkstra’s shortest path algorithm [28]. Let $\mathcal {E}$ denote the set of shortest paths $d_{ii^{\prime }}<\infty $. When the interaction dataset *P* is quite sparse, we choose a larger PPI dataset of the same species, whose gene set is a superset of $\mathcal {I}$. Use of a larger PPI dataset incorporates additional biological hints for the analysis. By choosing the shortest path as a potential PPI, we obtain a higher confidence in deriving inferred implicit interactions. We also avoid the need to consider many paths by introducing all the transitive PPIs. For example, we can avoid the hassle of considering protein interactions in a clique.

#### Scoring the shortest path

Implicit interactions are designed to have lower scores compared to explicit interactions; scores are assigned inversely proportional to the number of intermediaries, i.e., implicit interactions with a larger number of intermediaries have proportionately lower scores. The path length $d_{ii^{\prime }}$ indicates the strength of the implicit association between gene *i* and *i*^′^. Let the confidence score *c*_*d*_∈[0,1] be a non-increasing number series over path length *d*, which quantifies the implicit protein interaction between *i* and *i*^′^. Clearly, *c*_1_ should be 1 for 1-hop connections. For *d*≥2, we set *c*_*d*_=*ζ*^*d*−1^ where *ζ* is the probability of extending the path by one hop. We assume that each additional hop is independent of the previous hop.

Using the equivalence assumption, we deduce the closed form approximation for *ζ* as follows. Suppose there are paths of length *d*=2,…,*∞* between proteins *i* and *i*^′^, then there should be a direct interaction between protein *i* and *i*^′^, i.e., $\sum _{d=2}^{\infty } c_{d}=1$. Thus, the solution to this infinite summation $\sum _{d=1}^{\infty }\zeta ^{d}=\frac {\zeta }{1-\zeta }=1$ is *ζ*=0.5. We therefore assign a confidence score of *c*_*d*_=0.5^*d*−1^ to each shortest path of length *d*. This score decreases exponentially with the number of hops, e.g., *c*_2_=0.5,*c*_3_=0.25,*c*_4_=0.125, etc.

For a gene pair (*i*,*i*^′^) in $\mathcal {I}$ with a corresponding shortest transitive path length $d_{ii^{\prime }}$, a confidence score of $c_{d_{ii^{\prime }}}$ is assigned omitting $\sum _{pl>d_{ii^{\prime }}}^{\infty } 0.5^{pl-1}$. In fact, this confidence score is a conservative estimate of the real confidence score because it assumes that there exists path lengths of length *d*>*pl*. In practice, there may exist only one path between two genes. The shortfall is at most $\sum _{pl=d>2}^{\infty } 0.5^{pl-1}<0.5$. If there exist many paths of varying lengths (overwhelming evidence) between a genes pair, then the confidence score becomes an underestimate. On the other hand, if there exists only one path, which is also the shortest path, then the confidence score is an accurate depiction of the strength of regulation between two genes.

We use the method of [27] to estimate PPI scores by modeling protein interactions as a function of two random variables: (1) the implicit PPI confidence score based on the shortest path and (2) the Pearson correlation coefficient of expression measurements for the corresponding genes. Unlike the approach of [27], which learns the weight of each random variable from a training set of positive and negative examples, we simply use uniform weights. Our formulation involves only two variables: the implicit PPI confidence score and Pearson correlation coefficient derived from GE data. Let *W* with elements *W*_*ij*_ denote the graph with connection strengths computed from these two variables, then

6 $$  W_{ij} = \rho_{ij} + c_{d_{ij}} = \rho_{ij} + 0.5^{d_{ij}-1},  $$

where *ρ*_*ij*_ is the correlation of gene *i* and *j* in the gene expression data, and *d*_*ij*_ is the shortest path length between gene/protein *i* and *j* in the PPIN.

Until now, *W* collected the evidence from GE correlation and transitive protein-protein interaction for predicting direct gene-gene interactions. In fact, there are other evidences for gene-gene interactions. Similar to [29] that used a random walk model to consider the incompleteness of current gene ontology (GO) or PPIN evidences, we propose a random walk model to allow collecting additional evidence in a random fashion for predicting gene-gene interactions.

#### Topological connectivity via random walk transitions

The random walk models including Markov clustering algorithm [30] and PageRank [31] have been successfully used to model the link structure of graphs. Likewise, we extract the topological structure of the extended PPIN by using a random walk model instead of modelling with a small-world clustering coefficient [27].

Given an undirected graph *W*, random walk transition matrix *T* is defined as

7 $$  T= \begin{cases} c \frac{W_{ij}}{\sum_{j=1}^{I} W_{ij}} + \frac{1-c}{I} & \text{if \(W_{ij}\neq 0\)}, \\ \frac{1-c}{I} & \text{otherwise}. \end{cases}  $$

where *c*∈(0.5,1) is the fusion parameter (a.k.a. the damping factor in PageRank, typically set to 0.85) that determines the probability of the next transition from one of the outgoing links versus the transition from going to any random link.

Enright et al. [30] has shown that transition matrix *T* converges quadratically to an equilibrium state representing the topological connectivity of the graph. The converged matrix, denoted by $\hat {T}={\lim }_{k\rightarrow +\infty }T^{k}$, can be computed by Markov CLustering (MCL) algorithm [30]. In our context, the converged matrix of extended PPIN shows how likely protein pairs are related to one another.

#### Extended PPIN

Recall that the confidence score of how likely the predicted PPI occurs are inferred from the shortest path evidences from existing database, which can be treated as the confidence from biological knowledge. Here, the topological connectivities of the extended PPIN are inferred from the graph structure or the topological properties by random walk transitivity. We combine both of them to refine the confidence score of how likely all protein pairs will interact with each other in the PPIN as *C*=*W*+*W*∗*T*.

Combining *W* from (6) and the transition matrix from (7), the final confidence PPI scores are arrived as follows.

8 $$  C_{ij}=0.5\times W_{ij}+0.5\times\sum_{k=1}^{I} W_{ik}\hat{T}_{kj},  $$

where *W*_*ij*_ denotes the confidence score derived from the PPIN database and GE correlations and $\hat {T}_{kj}$ represents the converged random walk probability. Hence, $W\times \hat {T}$ yields the confidence on the link structures of the original network. The summation term actually compensates for the confidence score *W*_*ij*_ that could have been underestimated in the previous step using the shortest path.

To summarize, score *C*_*ij*_ starts from the values estimated from PPIN and GE correlations and then updated with converged values of a random walk model. Thus, the confidence score takes into account topological properties of GRN. The extended PPIN is derived from thresholding the C scores.

### Gaussian hidden Markov model

When genes collaborate to achieve a specific task, the corresponding protein products generally interact [13]. The PPI serve as valuable hints to underlying regulations among genes in GRN pathways. To fuse PPI and GE data, we treat gene expressions as observations, pathways as hidden states, and protein interactions as transitions in a Gaussian Hidden Markov Model (GHMM) [24]. In the GHMM, gene expression is the observed variable, the pathway it belongs to is the hidden state, given a hidden state (pathway), the expression of genes in the pathway follows a Gaussian model. The are gene-gene interactions are treated as transitions between their corresponding hidden states (pathways): if they are in the same pathway, the gene-pathway assignments are reinforced; otherwise, they are penalized. In this way, the GHMM combines the extended PPIN in the gene-pathway assignments.

**Fig. 2:**
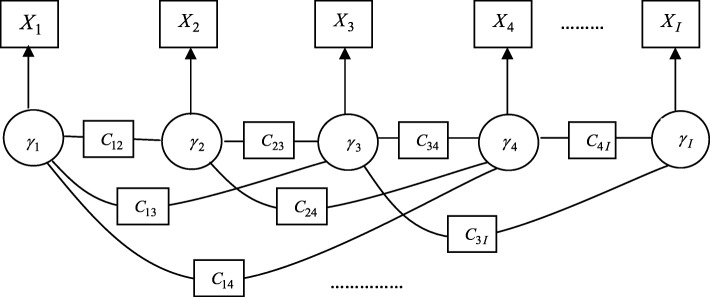
A sample Gaussian Hidden Markov Model (GHMM) model. The gene expression observation of gene *i* is denoted by *X*_*i*_, the circles denote hidden variables *γ*_*i*_, and PPI confidence scores between genes *i* and *j* are denoted by *C*_*ij*_

Hidden Markov random field (HMRF) models assume that the conditional distribution of a variable obeys Markov property, i.e., the probability of a variable only depends on the neighbouring variables (see [32] for a complete description of HMRF). In the present context, HMRF graph is represented by a set of nodes where node *i* represents observation *x*_*i*_ with hidden variable *γ*_*i*_ and the neighborhood graph is represented by constraints *C*_*ij*_, each of which indicates the edge weight between node *i* and node *j*. Specifically, the extended PPIN with confidence score *C* is used as constraints (prior knowledge) to the GHMM, i.e., they are considered neighbourhood structural priors of the corresponding gene-gene associations. Figure 2 shows a sample GHMM model with PPI priors.

The prior probability of a particular cluster assignment *Γ* follows a Gibbs distribution [33] as $p(\Gamma |C)=\frac {1}{Z}\exp \left (\sum _{i=1}^{I}\sum _{j\neq i}^{I} -C_{ij}\delta (\gamma _{j}\neq \gamma _{i})\right)$ where *δ*(·) is the indicator function and $Z=\sum _{\Gamma }p(\Gamma |C)$ is the normalizing function, and *C*_*ij*_ is the PPIN confidence score between gene *i* and *j*. Exact inference of the posterior requires the complete evaluation of $p(\Gamma |\mathcal {X},C) \propto p(\mathcal {X},\Gamma)p(\Gamma |C)$, where $p(\mathcal {X},\Gamma)$ follows the Gaussian mixture model defined in (1). The posterior [34] probabilities for the multivariate Gaussian case are approximated as

9 $$ \begin{aligned} & \tau_{il}=p(x_{i},\gamma_{i}=l|C) \\ = &\frac{\theta_{l} p(x_{i}|\mu_{l},\Sigma_{l})}{\sum_{l^{\prime}}^{L} \theta_{l^{\prime}} p\left(x_{i}|\mu_{l^{\prime}},\Sigma_{l^{\prime}}\right)}\exp\left(\sum_{j\neq i}-C_{ij}(1-\tau_{jl})\right), \end{aligned}  $$

where *θ*_*l*_ is the mixture weight and *p*(*x*_*i*_|*μ*_*l*_,*Σ*_*l*_) is the probability density function of the component model.

Based on the above, we learn a GHMM by using component-wise EM algorithm of “Soft-Clustering of GE by Gaussian mixture model” section, from which we derive the GRN from the pathway assignments: *G*=*τ*×*τ*^*T*^. The gene interaction score is then refined as *R*=0.5×*G*+0.5×*C* where *C* is the confidence score of PPIN in (8).

### Bayesian Gaussian network model

The GHMM assumes that the genes in a pathway are mutually and conditionally independent and do not consider partial correlation of their mRNA expression levels. To model this inter-gene dependency in a pathway, we use a Bayesian network (BN), a directed acyclic graph with local conditional distributions. Here, nodes represent genes, proteins, and/or metabolites, while edges represent molecular interactions such as protein-DNA and protein-protein interactions, including indirect relationships like those from inferred PPI.

BN are prone to overfitting of noisy or sparse training data such as gene expression data. Overfitting could lead to a vastly incorrect graph structure of GRN. Furthermore, since protein levels are unobservable from microarray data, vast majority of BN models of GRN proposed so far only include mRNA levels of genes as nodes but do not include protein levels. Thus, one way to reduce overfitting the BN is to incorporate the prior knowledge extracted from PPI. We thus propose a novel scoring scheme to feed implicit protein interactions (derived from GHMM) as structural priors into BN, thereby enhancing the robustness of predicted GRN.

Another major limitation of BN is its exponentially growing solution space with respect to the size of the network and thus approximate solutions such as those using Monte Carlo Markov Chain (MCMC) or genetic algorithms (GA) have been used. Such solutions are prone to errors and suboptimal. Since gene regulations exist among the genes in the same regulatory pathway, we constrain the parent set of each gene in the BN by first detecting the pathways, thereby greatly reducing the solution search space for subsequent computations. In a GRN, every gene appears in multiple pathways and therefore the relationship between genes in each pathway cannot be solved independently, i.e., by modelling each pathway as an independent BN. We thus use a Bayesian Gaussian Mixture (BGM) [35] that simply implements a BN with Gaussian Mixture observations. Here, observations for the Gaussian mixture reflect experimental observations, i.e., the experiments/observations are hard-clustered by using Gaussian Mixture Models. We thus use a MCMC inference method to learn the BGM model with structural priors derived from R scores in “Gaussian hidden Markov model” section, as shown in Fig. 1.

The BN is defined by a graph $\mathcal {G}$ with a family of conditional probability distributions and their parameters ${\mathcal {Q}=\{q_{i}\}}$, which together specify the joint distribution over the variables $p\left (\mathcal {X}|\mathcal {G},\mathcal {Q}\right)$. The joint distribution in a static BNs is factorized as $ p\left (\mathcal {X}|\mathcal {G},\mathcal {Q}\right)=\prod _{i=1}^{I} p(x_{i}|\pi _{i},q_{i})$ where each node *x*_*i*_ depends only on its parent nodes *π*_*i*_. The parameter matrix $\mathcal {Q}$ is comprised of *I* vectors where each vector *q*_*i*_ specifies a local probability distribution.

If we assume a Gaussian Bayesian Network (GBN), the parameter vector $q_{i}=\left \{\mu _{\pi _{i}},\sigma _{\pi _{i}}\right \}$ consists of the mean and standard deviation of the local probability distributions $p(x_{i}|\pi _{i},q_{i})\sim N\left (\mu _{\pi _{i}},\sigma _{\pi _{i}}\right)$. If we assume conditional independence among the *J* experiments, then $p\left (x_{i}|\pi _{i},q_{i}\right)=\prod _{j=1}^{J} p\left (x_{ij}|\pi _{i}=x_{\pi _{i},j},q_{i}\right)$ where $p\left (x_{ij}|\pi _{i}=x_{\pi _{i},j},q_{i}\right)\sim N\left (\mu _{\pi _{i},j},\sigma _{\pi _{i},j}^{2}\right)$. Assuming parameter independence, prior distribution $p(\mathcal {Q}|\mathcal {G})$ of the unknown parameters is expressed in terms of *I* local prior distributions: $p(\mathcal {Q}|\mathcal {G})=\prod _{i=1}^{I} p(q_{i}|\mathcal {G})=\prod _{i=1}^{I} p(q_{i}|\pi _{i})$. The marginal likelihood $p(\mathcal {X}|\mathcal {G})$ is thus the integral over the parameter space:

10 $$ \begin{aligned} p(\mathcal{X}|\mathcal{G}) &= \int p(\mathcal{X},\mathcal{Q}|\mathcal{G}) d\mathcal{Q} \\ &= \int \left(\prod_{i=1}^{I} p(x_{i}|\pi_{i},q_{i}) p(q_{i}|\pi_{i})\right) d \mathcal{Q}, \end{aligned}  $$

which can be rewritten as $p(\mathcal {X}|\mathcal {G})= \prod _{i=1}^{I} \Psi (x_{i},\pi _{i})$, where $\Psi (x_{i},\pi _{i})=\int p(q_{i}|\pi _{i})p(x_{i}|\pi _{i},q_{i}) d q_{i}$.

GE data are generated under a variety of conditions that may include time-series experiments, i.e., each experiment may be a continuation of preceding experiments. Thus, it is infeasible to assume independence among individual experiments. Taking a step back, we assume the experiments are generated from Gaussian mixtures and adopt a Bayesian Gaussian mixture model to represent GE data.

Suppose there are *K* mixtures {*D*^(*k*)^} where each mixture *D*^(*k*)^={*x*_·,*j*_}_*I*∗*m*_ represents some *m* attributes of a gene set $\mathcal {I}$, and {*θ*_*k*_} mixture weights of experiments in the BGM, then we express

11 $$ \begin{aligned} p(\mathcal{X}|\mathcal{G}) & = \sum_{k=1}^{K} \theta_{k} p\left(D^{(k)}|\mathcal{G}\right) \\ & = \prod_{i=1}^{I} \sum_{k=1}^{K} \theta_{k} \Psi\left(D^{(k)}, x_{i},\pi_{i}\right), \end{aligned}  $$

where mixture weights {*θ*_*k*_} are estimated with the EM algorithm, assuming independence among genes. Theoretical considerations and more details specifying the GBN parameters estimation can be found in [36].

#### Constraining the set of parent candidates

The GHMM derived in “Gaussian hidden Markov model” section can provide some structural constraints to BN learning. First, parents of genes should hail from the same regulatory pathway, i.e., if gene *i* belongs to some regulatory pathways, then other genes in these regulatory pathways could be its potential parents. In this manner, we generate a more accurate parent candidates set for each gene in addition to using the relationship defined in GRN. This is a nice middle ground between an exhaustive search through all the genes for potential parents and the limited set of GRN derived parents.

With the refined confidence score *R* assigned to every gene pair, the parent candidate set is limited to only top-*k* neighbours or top-*N* gene pairs as determined experimentally. One simple way to determine *k* and *N* is to use the average number extracted from a real GRN having a comparable size.

#### Incorporating neighborhood confidence scores

From [33], the prior probability of a particular structure $\mathcal {G}$ with constraints *R*={*R*_*ij*_} follows a Gibbs distribution,

12 $$ p(\mathcal{G}|R)=\frac{1}{Z}\exp\left(\sum_{i=1}^{I}\sum_{j\neq i}^{I} -R_{ij}\left(1-\mathcal{G}_{ij}\right)\right)  $$

where $Z=\sum _{\mathcal {G}}p(\mathcal {G}|R)$ is a normalizing function.

By using Markov networks framework to represent the correlations between neighbouring links, joint probability of data $\mathcal {X}$, given the BGM structure $\mathcal {G}$ and constraints *R*, can be written as $p(\mathcal {X},\mathcal {G}| R)= p(\mathcal {X}|\mathcal {G})p(\mathcal {G}|R)$. That is,

13 $$ {\begin{aligned} p(\mathcal{X},\mathcal{G}| R) & = \frac{1}{Z}\prod_{i=1}^{I} \left[\sum_{k=1}^{K} \theta_{k} \Psi\left(D^{(k)}, x_{i},\pi_{i}\right)\right]\exp\left(\sum_{j\neq i}^{I} -R_{ji}(1-\mathcal{G}_{ji})\right) \end{aligned}}  $$

#### Learning BGM with constraints via MCMC

Given GE data $\mathcal {X}$, fusing association scores *R* computed from GHMM and fixed parameters $\mathcal {Q}$, the structural posterior probability is written as

14 $$ \begin{aligned} p(\mathcal{G}|\mathcal{X}, \mathcal{Q}, R) & = \frac{p(\mathcal{X},\mathcal{G}| \mathcal{Q}, R)} {p(\mathcal{X})} \\ & \propto p(\mathcal{X},\mathcal{G}|\mathcal{Q}, R) \end{aligned}  $$

In the context of static BNs, different MCMC methods have been proposed for sampling Directed Acyclic Graphs (DAG) from the structural posterior distribution. We adopt the structural MCMC approach of [35] to sample our BGM structure $\mathcal {G}$ from posterior distributions $p(\mathcal {G}|\mathcal {X}, \mathcal {Q}, R)$. The idea is to give preference to the structures of higher posterior probability. The details of MCMC method to learn DAG from BGM are described in the supplementary materials. With model averaging [37], i.e., we run MCMC structural learning for a maximum number of iterations and each predicted edge in the GRN is assigned a confidence score that is measured by the number of occurrences of predicted edges among the generated graphs.

## Experiments and results

### Datasets

Our methods were tested on a dataset consists of 25 yeast genes, similar to those used by similar studies on fusion of GE and PPIN [6, 16]. The only difference is that we added five more genes (CDC28, CLB6, CLN3, FUS3, FKH2) that are highly connected to the 25 genes, based on current biological databases and literature. The 30 genes network involves in the cell-cycle regulation of yeast. A cell cycle is comprised of four phases: (i) Gap 1 (G1) phase - the checkpoint to ensure that the cell is ready for division, (ii) Synthesis (S) phase - involving DNA replication, (iii) Gap 2 (G2) phase - a checkpoint to ensure that the cell is ready to enter the next phase, and (iv) Mitotic (M) phase referring to cell division.

We collected data from several resources in order to construct a comprehensive target ground-truth network: (i) GeneNetWaver (http://wwwmgs.bionet.nsc.ru/mgs/gnw/genenet/) [38, 39] that includes 12873 transcriptional regulations among 4441 yeast genes where 62 transcriptional regulations are among our 30 cell cycle genes; (ii) 141 literature-reported regulatory relations among the 30 target genes manually collected from [40, 41], etc.; and (iii) Lee et al. [42] which proposed a integrated functional association score from mRNA expression data, PPIN, and literature mining edges of 5552 yeast genes. The linkage score showed good performance on independent benchmark datasets from KEGG (https://www.genome.jp/kegg/), STRING (https://string-db.org/), Gene Ontology (http://www.geneontology.org/), and experimentally-determined subcellular localization. We collected the “IntNet” from [42] with a high likelihood of regulation score above 0.5, which included co-expression regulations from 717 experiments for yeast (divided into 27 experimental categories), protein-protein interaction experiments, and literature mining of edges. From this network, 166 linkages are among the 30 target genes. In total, the ground-truth network has 317 regulations among 30 cell cycle genes.

The GE data was obtained from [43] that contains 77 experiments collected over 8 yeast cell cycles by using four different synchronisation protocols. The PPI data for *Saccharomyces Cerevisiae* was downloaded from BioGRID (http://thebiogrid.org/), that contains 6263 proteins and 210,996 interactions.

For comparison, true positives (TP), false positives (FP), and false negatives (FN) of edges were computed by comparing the predicted pathways to the target ground truth network. Various performance metrics including Precision, Recall, and F1-score were evaluated. To compare with state-of-the-art methods of predicting GRN from GE data, such as CLR [26], GENIE3 [44], TIGRESS [45], we determined AUROC (The area under the receiver operating characteristic (ROC) curve) and AUPR (The area under the precision-recall (PR) curve) scores as defined in [44]. For these metrics, the best results that are significantly different (*p*-value < 0.05) from other methods are shown in bold. We implemented *t*-test to check whether the best result is significantly different from other methods; If the best result is not significantly different from the next best ones, we choose the best results as one group and the others as another group and then implemented the unpaired t-test for the two groups.

### Feasibility of extending PPIN

In “Structural inferences from PPIN” section, we described a method to extend PPIN, using transitive protein interactions and assigned confidence scores C to the predicted protein interactions. We will demonstrate the feasibility of predicting protein interactions from this extended PPIN in this section.

To test how well extended PPIN (derived from C scores) recover an incomplete PPIN, we randomly selected 200 yeast genes and assign 3008 PPIs from BioGRID among them. We used 10-fold cross-validation on the PPINs of these 200 randomly selected genes, by randomly removing 10% edges in the target PPIN, deriving extended PPIN based on the remaining 90% edges, and then comparing the extended PPIN with the target PPIN on the missing 10% edges. We repeated 10-fold cross-validation experiments for 10 times to show the robustness of the method. The results demonstrated that the extended PPIN (when the cut-off threshold for C score is set as 0.5) effectively recover 346 out of the 383 removed edges on average. Further, our extended PPIN based only on 90% PPIN edges predicted the entire PPIN with an average F1-score of 43%, indicating the effectiveness of the method of extending PPIN (C score) and in its utility in predicting the missing PPIN.

As BioGrid may contain functional linkages predicted as protein-protein interactions, we also choose another PPIN dataset to show the reliability of the method of extending PPIN (C score) to predict real PPIs. The yeast PPIN data from http://interactome.dfci.harvard.edu/S_cerevisiae consists of the full set of physical interactions that occur in a physiologically relevant dynamic range between all its macromolecules, including protein-protein, DNA-protein, and RNA-protein interactions. By combing all the physical interactions, co-complex membership associations [46] and literature-curated interactions, [47] collected in “Yeast Interactome Datasets” from which we get 11995 interactions of 2234 yeast proteins where only 100 PPIs are found among the same randomly selected 200 yeast genes. The experiments of 10-fold cross-validation on these 100 PPIs showed that our extended PPIN (when the cut-off threshold for C score is set as 0.55) effectively recovers the removed edges on average. It also shows that the extended PPIN based only on 90% PPIN edges predict the entire PPIN with an average F1-score of 36.62% (average precision at 39.56% and average recall at 34.10%). This further validates the reliability of the method of extending PPIN (C score) to predict the real PPIs.

In the BGM with prior model, we added the prior information into the likelihood of gene expression data of the learned BN structure. If the GRN learned from GE data is consistent with prior information, the likelihood is reinforced; otherwise, the likelihood is penalized. Thus, the GRN from the fusion model achieves the maximum consistency between gene expression data and prior information. We choose the informative BioGRID PPINs as priors in our fusion model.

**Fig. 3:**
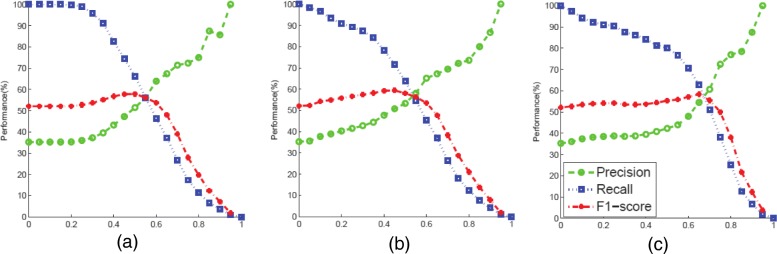
Performance comparison of C and R scores from the subnet or the global information at various cut-off thresholds on 30 yeast genes ground-truth network. **a** C scores (global) **b** C scores (subnet) **c** R scores (subnet)

In order to show the effectiveness of predicting gene regulations from extended PPIN, we evaluated the extended PPIN alongside the original PPIN (denoted by “raw PPIN”). As mentioned in “Structural inferences from PPIN” section, we can extend PPIN either from a sub-network or from the global network where the global network is the PPIN for the whole genome of the same organism and the sub-network indicates the subnet PPIN defined by the genes in the target gene set. When the PPIN was extended from subnet information, we only consider transitive links among the target gene set. If the subnet PPIN is quite sparse, we choose the global network to make use of transitivity. However, extended PPIN from global information may introduce more noise especially when PPIN is spurious. We experimentally compared differences in the performance of extending PPIN from the sub-network (i.e., 30-gene PPIN for the benchmark) and the global network (i.e., the complete PPIN from 6263 yeast genes) on the 30-gene benchmark network. Table 1 shows the performance with three PPINs: raw PPIN, extended PPIN with subnet information, and extended PPIN with global information on predicting the GRN of the 30 benchmark genes. The cut-off thresholds to generate GRN from C score for the extended PPINs were set as 0.5 for a better trade-off between precision and recall as seen in Fig. 3.

**Table 1:** Performance of predicting GRN with different PPINs on 30 yeast genes ground-truth network

Method	*TP*	*FP*	*FN*	*Prec.(%)*	*Rec.(%)*	*F1(%)*	*AUROC*	*AUPR*
Raw PPIN	165	95	152	63.46	52.05	57.19	0.698	0.591
Extended PPIN (subnet)	229	220	88	51.00	**72.24**	59.79	**0.713**	0.533
Extended PPIN (global)	240	260	77	48.00	**75.71**	58.75	0.696	0.500

Best performance measures that are significantly different are shown in bold

Comparing two extended PPINs, we see that the extended PPIN with global information outperforms the prediction with only subnet information on recall, sacrificing some precision. This indicates that global information may introduce more incoherent genes to make prediction noisy. The observation that both local and global information achieve comparable performance shows that global information can complement subnet information from sparse PPIN data. Clearly, if not much protein interactions are lost in the target PPIN, extended PPIN with subnet information is a good choice to balance the precision and recall, as illustrated by the best F1 performance with extended PPIN from local information. We thus choose extended PPIN with subnet information for the 30-gene benchmark network in subsequent experiments.

Comparing extended PPIN with the raw PPIN, we see that the extended PPIN with subnet information yielded a notable 20% improvement on recall compared to raw PPIN at a hefty decrease of 12% in precision. This shows the feasibility of deriving regulations by using the transitivity present in the PPIN. It is reasonable that raw PPIN predicts GRN with the highest precision, indicating how much protein interactions in the current PPIN database are consistent with gene regulation data. The extended PPINs provide better recall/coverage on predicting GRN even with the subnet information, giving the best F1-score, indicating the incompleteness of the current PPIN database. The precision of extended PPIN can be improved by raising the C score threshold but the recall will then be decreased at the same time. Since our approach is a coarse-to-fine framework to predict GRN where the extended PPIN is used to limit candidate gene interaction set (coarse step), a higher coverage or recall is preferred. As such, we choose 0.5 as the cut-off threshold that gave us a reasonable recall. The high false positive rate from the extended PPIN can be reduced in the subsequent “refinement” step. As subsequent experiments demonstrate, our methods predict the final GRN with a high precision. Therefore, what follows next are demonstrated only with extended PPIN data.

### Comparison of performances

We benchmarked the generated GRNs against the ground-truth networks. All the methods except the BGM generated networks with undirected edges. When comparing an undirected graph to a directed one, we considered an existence of a matched link regardless of its direction. For comparison, all the networks generated by the BGM were sampled and averaged for 10 models each of which was learned from 1000 iterations of MCMC structural moves. The performance of the BGMs were further improved by increasing the number of models by model averaging and the iterations of structural moves.

Since the generated graphs from C, R, and *G* assigned confidence scores for the edges, i.e., probabilities of edge existence, we chose a cut-off threshold and constructed the GRN, based on the scores: if the edge score is higher than this threshold, we confirm the existence of an edge; and otherwise, the edge was rejected. From experiments, as seen in Fig. 3, we found that the best cut-off thresholds (for C and R scores) that maximize the F1-score (which is also the best trade-off between precision and recall) of GRN tend to be clustered around 0.5. For GRN structure $\mathcal {G}$ from clustering or the BGM, scores came from the probability of genes belonging to the same pathway or the model averaging where both reflected how likely genes interact. We therefore intuitively fixed 0.5 as the threshold to determine interaction edges. Thus, for consistency, we set the cut-off thresholds for all our graphs to be 0.5. The confidence scores were normalized between 0.0 and 1.0 before averaging. The thresholds can also be experimentally fine-tuned to further improve the performances of our methods. The vast majority of existing works on prediction of GRN choose the cut-off of the confidence score as a trade-off between sensitivity and specificity of prediction [48].

Table 2 compares performances of building GRNs by various methods. The first four methods (CLR, GENIE3, TIGRESS, GMM) are recent state-of-the-art methods of predicting GRN from GE. We use MATLAB implementations of CLR [26], GENIE3 [44], and TIGRESS [45]. The other three methods (GHMM, R scores from GHMM, BGM with R scores) are fusion methods that use both the GE and PPIN data as described in the “Methods” section. As seen, all three fusion methods significantly outperformed (5–20% better in all metrics) the methods using GE data only.

**Table 2:** Performances of prediction of GRNs by various methods on 30 yeast genes ground-truth network

Method	*TP*	*FP*	*FN*	*Prec.(%)*	*Rec.(%)*	*F1(%)*	*AUROC*	*AUPR*
CLR	190	312	127	37.85	59.94	46.40	0.555	0.388
GENIE3	128	202	189	38.79	40.38	39.57	0.546	0.395
TIGRESS	140	207	177	40.35	44.16	42.17	0.546	0.392
GMM	172	266	145	39.27	54.26	45.56	0.583	0.412
GHMM	258	329	59	43.95	**81.39**	57.08	0.664	0.461
R scores (GHMM)	250	262	67	**48.83**	78.86	**60.31**	**0.705**	**0.501**
BGM (R scores)	202	237	115	46.01	63.72	53.44	0.627	0.446

Best performance measures that are significantly different are shown in bold

To show the effect of extending PPIN by incorporating transitive edges, we also compared three fusion models with and without extending the PPIN (i.e., by using only raw PPIN data). The three fusion methods using extended PPIN outperformed the methods using only raw PPIN. This demonstrates the effectiveness of using extended PPIN in GRN inference.

The GRNs obtained using GHMM and refined R scores are undirected graphs, indicating gene interactions but not specifically the directional regulations. To figure out the directional dependencies among genes, a directional model such as BGM is needed. The BGM model based on R scores as constraints in fact gave the overall best performance and thus deserves further investigation.

### BGM with different priors

Using the same set of parent candidates and the BGM model, we evaluated whether the BGM using refined confidence scores R can hold its own when benchmarked against other PPIN fusion methods. Specifically, we compared its performance with two well-known methods. Nariai et al. [14] tested if each added protein pair form a protein complex by considering the complex as a virtual node in the network. The method computes the principal component analysis (PCA) model of the protein pairs from original observations and checks if the protein complex contributes to a higher likelihood. If a higher likelihood is obtained, the complex (pair) is accepted and otherwise rejected. Imoto et al. [15] updated PPIN whenever the learned GRN produced different structure. Each inconsistent edge in the learned DAG is perturbed by either removing or reversing its direction. If a perturbation leads to a better likelihood, the perturbed PPIN is accepted otherwise it is rejected.

Both methods needed significantly more operations than the basic models and increased already substantial computational complexity. Further, they tend to converge to local optima given their greedy hill-climbing nature. Nariai’s method can only increase the protein interactions contributed by a protein complex if the PCA projection of the complex performs better than using raw data. Imoto’s method is overly-optimistic as it assumes that the learned DAG is correct. However, the MCMC method is known to only simulate a DAG; it may accept a large number of incorrect edges in one iteration and could accept all of them as updated PPIN in the subsequent iteration. Hence, updating the PPIN based on a learned DAG is unreliable.

To show the utility of each component in the fusion model, we compared the performance of the BGM by using priors from the GMM only, the PPIN only or the GHMM, as shown in Table 3. For the priors from PPIN, we also tested two existing fusion methods with the same priors. We described different components of our method as follows: “BGM (GMM)” denotes the BGM with parental constraints from GMM and predicts GRN only from GE data without PPIN data; “BGM (C scores)” denotes the BGM constrained with C scores from extended PPIN; “Nariai et al. (GHMM)” and “Imoto et al. (GHMM)” use parental constraints from the GHMM with extended PPIN to learn GRNs in BGM with corresponding fusion method; “BGM (R scores)” uses the extended PPIN in the GHMM fusion model and then R constraints for the BGM. For comparison, we also included a BGM without any constraints as the baseline.

**Table 3:** Performance of GRNs generated from the BGM with different priors on 30 yeast genes ground-truth network

Method	*TP*	*FP*	*FN*	*Prec.*(%)	*Rec.*(%)	*F1*(%)	*AUROC*	*AUPR*	*Time*(sec)
BGM	139	201	178	40.88	43.85	42.31	0.553	0.390	636
BGM (GMM)	185	293	132	38.70	58.36	46.54	0.555	0.383	606
BGM (C scores)	170	150	147	**53.13**	53.63	**53.38**	**0.653**	**0.490**	**518**
Nariai et al. (GHMM)	176	273	141	39.20	55.52	45.95	0.570	0.408	1174
Imoto et al. (GHMM)	184	293	133	38.57	58.04	46.35	0.565	0.401	1150
BGM (R scores)	202	237	115	46.01	**63.72**	**53.44**	0.627	0.446	608

Best performance measures that are significantly different are shown in bold

The results in Table 3 show that the priors from GHMM significantly improve TPs (highest recall) with comparable F1 scores (higher coverage at the slight expense of precision) compared to the methods using priors only from the PPIN. This indicates that GE clustering help to predict more TPs but at a lower precision. It also demonstrates that the use of the GRN structure from the GMM and extended PPIN in the prediction of GRN. These results further validate the utility of each component in the fusion model. Results in Table 3 show that with same structural constraints, the fusion BGM (with R scores) beats both the BGM proposed by Nariai et al. and by Imoto et al. This means that the R scores are effective measure of the reliability of PPIN edges. Table 3 also shows the running-time of different algorithms implemented with MATLAB on a machine with an Intel Xeon E5-1620 3.6GHz CPU and 8GB RAM. As seen, the priors given by our confidence scores lead to computationally more efficient procedures.

## Discussion & conclusion

We proposed an automated method to detect gene regulations by fusing GE data and PPI data. Gene expression data is first soft-clustered into regulatory pathways by a GMM where the number of regulatory pathways is automatically determined via a component-wise EM algorithm. Transitive protein interactions are derived using a novel confidence score and PPIN are refined and extended. The extended PPIN are then fused with the GMM derived from GE data, using a GHMM. This improves the biological relevance of the clustering results and also refines the confidence score of indirect gene regulations/protein interactions. Using refined PPI confidence scores, together with regulatory pathways obtained from the GHMM as structural constraints, a BGM model was used to capture direct gene regulations by using an effective and efficient MCMC procedure. Fusion of the BGM model with GHMM as structural priors generated more accurate GRN compared to those produced by the GHMM and the BGM models. We have experimentally shown that our procedures are more effective than two existing well-known methods for fusing GE and PPIN data.

Furthermore, the fusion framework reduces the overall time complexity of building GRN with Bayesian networks. The GHMM uses a component-wise EM algorithm to soft-cluster genes into pathways at a complexity *O*(*I*^2^×*L*^2^) (where *L* denotes the number of components and *I* denotes the number of genes), indicating that the method scales well on large networks (large *I*). The BGM structural constraints, i.e., the parent of each node must itself be a node from the same regulatory pathway, help to significantly reduce the search space, i.e., from *O*(*I*^*I*^) to approximately *O*((*I*/*L*)^*I*^). In fact, for larger *I*, the reduction can even be more pronounced.

Our approach shows the importance of systematically fusing multiple sources of biological evidences in inferring useful and reliable GRN. In fact, our framework can be extended to fuse more than two data sources, such as gene ontologies, biological pathways (KEGG), transcription factors (known regulators), information mined from literature, and other multi-omics data [4, 5, 10–12]. Our methods may be easily extended by further considering dependencies among the experiments [35] and are worth investigating further.

## Abbreviations

AUROC: The area under the receiver operating characteristic (ROC) curve
AUPR: The area under the precision-recall (PR) curve
BGM: Bayesian Gaussian mixture
EM: Expectation maximization
GHMM: Gaussian hidden Markov model
GRN: Gene regulatory networks
HMRF: Hidden Markov random field
MCL: Markov cLustering
MML: Minimum message length
MCMC: Monte Carlo Markov Chain
PPIN: Protein-protein interaction networks
